# The Relation of General Pathology to Medical Science

**Published:** 1885-04

**Authors:** 


### EDITORIAL DEPARTMENT.

### THE RELATION OF GENERAL PATHOLOGY TO MEDICAL SCIENCE.

Our readers must have certainly observed that so far as lay in our power we have endeavored to demonstrate the intimate relation existing betwen veterinary and human medicine, especially with reference to questions bearing upon public health. We intend to continue the work, and to endeavor to make this journal the JOURNAL OF COMPARATIVE MEDICINE not only in America but in the world. We cannot help expressing our gratification, a feeling we know our readers will coincide with, on hearing from so many sides "that we had succeeded in making it the best veterinary journal in the English language." With this we are not contented, however; it must be more than that; it must be accepted as an authority among the medical journals of the United States, and hence, so far as in our power, we intend to devote it to medical reform, and to the establishment of State medicine in the country, which means the inauguration of a national organization of public health officials, of which a competent veterinary police system is but a part. With reference to "medical reform," there is nothing more needed in the medical as well as in the veterinary schools, than that the graduated students should learn not only what disease is by its manifesting phenomena, but what it really is to the eye, and still more of what it essentially consists. In this regard we wish to call the earnest attention of our readers to the following remarks:

If we were to be asked, what is the greatest want in medical education in the United States? we should answer, "pathology." When one speaks of pathology to the average student of medicine, or even to the ordinary practitioner, his conception is always of that which can be seen by the organs of vision, viz., pathological anatomy. This is not the fact. True pathology is what one sees with the intellectual eye; pathological anatomy is but one of the steps, a most important one to be passed over before one can become a pathologist. Pathology, and we use the word always in its true, general, or universal sense, is the conclusion of all medical study. Pathology is the sum total of our knowledge of disease which finds its expression in intelligent practice. The aim of all true medical education should be to produce sharp observers and profound thinkers. The result of this combination is the really great practitioners. To the successful development of medical science it is absolutely necessary that there be a division of labor in the scientific as well as in the practical branches of our work. In the good old days the accumulations of knowledge and the methods of research bore no comparison to what the present practitioner has to contend with, and so we find the entire field of medicine commanded by one man, whom, without any disparagement, we could well name "the maid of all work" in medicine. This period found its culmination in the immortal Bœrhaave, the division of labor being introduced by those eminent men who formed the famous schools that went out from him, Haller, Gaubius and Van Surieten, each of whom, while retaining many of the characteristics of the master, represented in himself, to a varying degree, special work as physiologist, pathologist, or clinician.

Few realize the true meaning of the word "pathology." It is not pathological anatomy alone, as already said: it is the physiology of disease. Physiology is not the histo-anatomy of health any more than pathology is the patho-anatomy of disease. Physiology is the *how*, the reason *why*, of the action of the parts in health, as pathology is of the same in disease. Physiology is the philosophy of health, as pathology is of disease. Histology, whether normal or abnormal, is but the description of facts, conditions, open to ocular demonstration. Physiology and pathology are our explanation of these facts. Pathology is more than physiology, in that when one has completed his studies in anatomy, macro- and microscopy, chemistry, and physics, he can stop in his progress; no further preparatory study is necessary in the field of medicine in order that he may become a physiologist. Physiology has, however, its two great departments: the first, experimental and observational; the second, and greater, that which interprets the facts revealed by the former, the philosophical. The same is true of every other branch of science; each has had its men adapted to the detail work of experiment, observation, and recording of facts.

Then come the exceptional men—those intellectual phenomena who now and again, like comets, appear in the intellectual horizon. Bacon ushered them into the world, and Darwin, Virchow, Huxley, Tindall, Helmholz, Spencer and others have made grand the path of Inductive Philosophy. Such men are the true geniuses of the world; like the others, they are also sharp observers of facts; but beyond the sappers and miners in the field of scientific research, they possessed the powers of generalization; they gathered in the harvest which the others had reaped, and winnowing the chaff from the wheat, induced the general laws as indicated by the facts, and prepared the way for a new and younger corps of reapers to enter the field in further search for new facts.

I have indicated where the preparatory education of the physiologist could stop. Not so with the pathologist. He is the store-house of medical science. He must first become all that the physiologist is; he must acquire the most exact and extended knowledge of what health really is, not only in appearance, but in action, *i. e.*, its phenomena; also of the mysterious ways of development, and the variations from regularity which lead to the production of abnormality. It must be repeated, that without the most exact knowledge of what health is, it is absolutely impossible to have any idea of what disease is. I well remember the words of the greatest friend I ever had, and the teacher who has had the most influence on my life, as one day I was bothering with a pathological specimen: "My boy, throw that away; while with us, spend your whole time with the healthy organs; devote your entire energies to histology and physiology; hear the lectures, and gather all you can from the demonstrations in pathology; then when you leave us, go to Virchow, and there learn what disease is." Those words have never been forgotten. They led to the decision of my course in life.

To all students I again say, Learn what is normal, knowing that you will at once recognize the existence of abnormal conditions; you can thus become good pathological anatomists. Pathologists cannot be produced to order; they are born. Whether they become so or not, is largely due to the circumstances by which they are surrounded. But more: not only must the pathologist be able to read facts as they appear, and interpret their genesis—he must also have the ability to recognize their manner of manifestation, in order to make clear to the student their phenomena in life—that is, to be able to interpret their so-called practical value. Hence it is that even when his studies would appear as if completed with pathological anatomy, it is necessary that he have clinical experience, in order to become a clear thinker and practical teacher.

For my own part, I do not believe that the competent pathological anatomist, and far more the pathologist, can ever make a successful practitioner. He can, and should, become a sharp diagnostician, or even a great surgeon, but his knowledge of disease is such that he soon becomes so sceptical with regard to the value of medicines as to lose all confidence in them in given cases. He feels his own impotence in the presence of such diseases as caseous pneumonia, tubercular phthisis, or the malignant infectious diseases. To faith, a certain amount of ignorance is always necessary. If the pathologist is not, from the nature of his studies and intellectual constitution, suited for a practitioner himself, yet he is the necessary implement which we need, in order that our students shall become, as before said, sharp observers, clear thinkers, good diagnosticians, correct in prognosis, and hence trustworthy practitioners.

"The wheels of the gods grind slowly, but surely," and it now looks as if the pathologists were soon to be ground to dust under the accumulating load that research is gradually placing upon them. Once to be a pathologist it was only necessary that one knew man in health and disease. The birth of modern homo-pathology found its expression in Bichat, Laennec, Dudeytron and the French school of the first half of this century. The birth of the new school of pathology found its prophet in the greater master of our day, Rudolph Virchow. Virchow is the first of all the pathologists and patho-anatomists to whom the name of Comparative Pathologist can be given.

It is a grave mistake to call Veterinary, or Zoöpathology, "comparative." Whatever there is of it in existence—and there is little based upon exact research—is due to Virchow and his influence. The truth is that zoöpathological anatomy remains to be developed.

With the increased accumulation of the facts that have been thus far derived from these studies, the principles of general pathology induced from the study of disease in man, find again their confirmation, and we are more and more impressed with the fact that the laws of health and disease are the same wherever we find life.

Comparative Pathology is the study of disease, wherever found afflicting vitality. Not content with including the mammal world within its grasp, it now begins to extend still further, and we hear of studies of disease in fishes, birds, reptiles and even insects. With the accumulation of facts in these directions will come again the necessity of generalization, and the pathologists in the future will find their task still more difficult.

So far we have had only to do with life as connected with nerve-force, so-called animal life, in which there is more or less of a nervous organization. Research has long ago revealed to us that the axiom "Omnis cellula e cellula" could not be thus limited, however. This law was first laid in the study of the vegetable cell, and from thence generalized by Virchow to the animal world. We now find, to a complete comprehension of our work, that we must also extend our studies beyond the animate to the vegetable world, and that he who would become a perfect teacher must make himself acquainted with the general principles of vegetable anatomy and physiology. To the unreflecting student this may appear absurd, but let me assure him that the more general one's knowledge of the phenomena of life the better teacher can he become, and even the better practitioner. Would we could stop here; but further study has shown that vegetable life has also its diseases—that there is such a thing as vegetable pathology, under which lie certain general principles which cannot be ignored. To the sceptic on this point, as to its having any practical value, we would say: select some soft stemmed plant, out of which microscopic sections can be easily prepared, make a cut into it with a sharp knife, let it rest a day or two, inject into the substance of the stem, both above and below the cut, some colored fluid, or treat several stems in one way and another, and compare the microscopic sections with cuts from wound-healing in the mammalia, and you will be surprised at the analogy. There you will see exudation of serum, thickening of the stroma or frame-work, development of nuclei, proliferation of cells, and, if lucky, extenuation or development of the "soft canäle" or sap ducts. You will find the general principles, which have been taught you in your studies of man and animals, still more confirmed. It is for the botanist to collect the facts and to induce the laws they teach. The comparative pathologist has only to adapt them to the general principles of disease, where applicable.

Is our task done? With the study of disease, and as a branch of general pathology, we have still another subdivision to consider.

ETIOLOGY.

Once this was limited to man and his immediate surroundings; causes internal and external, but they did not extend beyond man. Research has already shown the intimate connection existing between many human diseases and our animal surroundings, either in the form of the food we eat, or as contagion. The study of anthrax and the fact of its communication to man again reveals the necessity of the pathologists having knowledge of the vegetable world. Bacillus Anthracis introduced to us the necessity of the study of that branch of scientific investigation known as mycology.

Pasteur chiefly led the way into this new world of research, while Koch, through the discovery of the value of solid culture media, has more completely developed it, and indicated the way we may profitably conduct the microscopic form of agriculture, and be enabled, without the aid of the microscope, to take the tares from the wheat in our miniature fields, and thus easily obtain "sein-culturen," where before the work was one of infinite labor and uncertainty. While with such germs as those of anthrax and phthisis, the most direct way of obtaining a pure culture is to inoculate a polluted one upon some susceptible animal, and then, with caution, from it upon some appropriate culture medium; by others, that cannot at present be so treated, we are oblige to follow the example of the husbandman and sow the tares and wheat together, then when both have ripened, we can easily separate them by the microscopic examination of the growths, and by studying the nature and appearance of the wheat in the media, soon learn to tell it from the tares, as the husbandman roots the weeds out of his field. He recognizes them by their appearance and manner of growth.

This is one of the secrets of Koch's method.

We have made but a beginning here. It would seem as if every form of bacterial life has some one medium in which it would develop in what is called a characteristic manner, though in others it might bear strong resemblances to other germs. This work requires much ingenuity in this direction, and with the discovery of new media, will be found new means of bacterial diagnosis with but the aid of the microscope.

For example, the celebrated "comma" develops in flesh-water gelatine, in the form of the lower half of a balloon, if the medium is suitably prepared, the basket being represented by the accumulation of the bacilli at the apex or point of the culture, the base being upwards. In blood serum it develops in the exact form of a blown out "cordon;" in both cases looking like a delicate veil in the media, the bacteria being at the bottom. Where developed on glass plates it has an infundibula or funnel-shaped form, being a small dark centre, surrounded by two rings of fluid medium of more or less opacity. In all these cases it readily develops in the ordinary room temperature in twelve to twenty-four hours.

The bacillus of tuberculosis, on the contrary, develops only with satisfaction in blood serum at a temperature of 38 to 40° C, and requires twelve to fourteen days to make its characteristic appearance. The entire work of bacterial study is largely of an empirical nature.

As regards tinction, some bacilli, as those of lupus and tuberculosis, and probably syphilis, have also their specific reaction, and the same will probably be found to be the case with numerous others as we discover and experiment with them.

It is necessary at this time to utter a word of warning against an error and danger that is gradually making its way into the minds of the younger members of our profession.

The hunt after and biological studies of bacteria is not the whole of etiology, nor by any means the fundament of preventive medicine.

Bacteria, or any other such cause of disease, are always to be looked upon as external causes.

The artificial culture of bacteria has shown us that, like the seeds the farmer sows, they will only grow on suitable ground. If the ground is not right, no development. Were this not so we should all die when exposed to infection, or at least become *sick*.

The predisposing causes, or internal conditions, those things which the study of pathology can alone reveal, have by no means lost their value by the discovery of the bacillus of anthrax, phthisis, cholera, or any other disease.

Another thing of equal importance, which must not be forgotten, is the nature of a contagious or infectious disease.

In most veterinary works, and many medical, the authors have so little knowledge of the logic of pathology as to be unable to distinguish between cause and nature.

It is not absolutely necessary to know what the cause is, in order to combat the extension of a contagious disease.

Our work is strengthened thereby and final prevention made more probable in some cases.

The prevention of glanders has not been made more possible by the discovery of Schutz and Lœfler, because we do not know where the bacillus originally develops, and only know that it is connected with the disease and can cause it by inoculation.

The same is true of tuberculosis.

But we do know how to get rid of glanders, because of the study of its nature, its manner of extension—and that by killing all known diseased horses we limit the possibility of extension to the smallest possible degree.

We know the cause of tuberculosis; we also know the nature of the disease. When we stop breeding weak-lunged children, when we stop the intermarriage of such, as well as consumptives, when our social relations become regulated by the principles of preventive medicine, we shall be able to combat the extension of phthisis.

The black death, bubo, pest, small-pox and rinderpest have become almost strangers to western Europe, because we have studied and learned their nature and their manner of extension, though we know nothing of their real cause.

With reference to the value of preventive inoculation as a general means of combatting diseases of a contagious nature, we are still in the very beginning of our studies.

Real prevention will, however, be best attained:

1st. By the study of the nature of disease and taking means to prevent its extension.

2d. By endeavoring to discover its real cause, studying its nature and endeavoring to prevent its development—a much harder task than the former.

This last branch of preventive study is only applicable, *in extenso*, to so-called malarial diseases, that is, such as find the conditions favorable to their development and the life of their cause in the conditions of the earth and air, or dwellings, drains, etc. Anthrax, intermittents, yellow fever, cholera, Texas fever and other such maladies are of this nature.

We have endeavored to outline the work of the general pathologist, and hope we have been able to create some respect for the "theorist" of medical science, in the minds of our readers. B.

